# Association of Tuberculosis With Household Catastrophic Expenditure in South India

**DOI:** 10.1001/jamanetworkopen.2019.20973

**Published:** 2020-02-12

**Authors:** Malaisamy Muniyandi, Beena Elizabeth Thomas, Nagarajan Karikalan, Thiruvengadam Kannan, Krishnan Rajendran, Balakrishnan Saravanan, Vikram Vohra, Tuoyo Okorosobo, Knut Lönnroth, Srikanth Prasad Tripathy

**Affiliations:** 1 Department of Health Economics, National Institute for Research in Tuberculosis, Indian Council of Medical Research, Chennai, India; 2 National Institute of Tuberculosis and Respiratory Diseases, New Delhi, India; 3 World Health Organization, Garki, Nigeria; 4 Department of Global Public Health, Karolinska Institute, Solna, Sweden

## Abstract

**Importance:**

The high household costs associated with tuberculosis (TB) diagnosis and treatment can create barriers to access and adherence, highlighting the urgency of achieving the World Health Organization’s End TB Strategy target that no TB-affected households should face catastrophic costs by 2020.

**Objective:**

To estimate the occurrence of catastrophic costs associated with TB diagnosis and treatment and to identify socioeconomic indicators associated with catastrophic costs in a setting where TB control strategies have been implemented effectively.

**Design, Setting, and Participants:**

In this cross-sectional study, 455 patients with TB in the Chennai metropolitan area of South India who were treated under the TB control program between February 2017 and March 2018 were interviewed. Patients were interviewed by trained field investigators at 3 time points: at the initiation of treatment, at the end of the intensive phase of treatment, and at the end of the continuation phase of treatment. A precoded interview schedule was used to collect information on demographic, socioeconomic, and clinical characteristics and direct medical, direct nonmedical, and indirect costs. Data analysis was performed from August 2018 to November 2019.

**Main Outcomes and Measures:**

Direct, indirect, and total costs to patients with TB. Catastrophic costs associated with TB were defined as costs exceeding 20% of the household’s annual income. A binary response model was used to determine the factors that were significantly associated with catastrophic costs.

**Results:**

Of 455 patients with TB interviewed, 205 (53%) were aged 19 to 45 years (mean [SD] age, 38.4 [16.0] years), 128 (33%) were female, 72 (19%) were illiterate, 126 (33%) were employed, and 186 (48%) had a single earning member in the family (percentages are based on the 384 patients who were interviewed through the end of the continuation phase of treatment). Sixty-one percent of patients (234 patients) had pulmonary smear positive TB. The proportion of patients with catastrophic costs was 31%. Indirect costs contributed more toward catastrophic cost than did direct costs. Multivariate logistic regression analysis found that unemployment (adjusted odds ratio, 0.2; 95% CI, 0.1-0.5; *P* < .001) and higher annual household income (Rs 1-200 000, adjusted odds ratio, 0.4; 95% CI, 0.2-0.7; *P* = .004; Rs >200 000, adjusted odds ratio, 0.2; 95% CI, 0.1-0.5; *P* < .001) were associated with a decreased likelihood of experiencing catastrophic costs.

**Conclusions and Relevance:**

Despite the implementation of free diagnostic and treatment services under a national TB control program, TB-affected households had a high risk of catastrophic costs and further impoverishment. There is an urgent demand to provide additional financial protection for patients with TB.

## Key Points

**Question:**

What are the costs incurred by patients with tuberculosis (TB) throughout the treatment period in a resource-poor country such as India, where diagnostic testing and TB treatment are provided free of charge?

**Findings:**

In this cross-sectional study of 455 individuals with TB, despite the implementation of free diagnostic and treatment services under a national TB control program, 31% of TB-affected households still experienced catastrophic costs.

**Meaning:**

These findings underscore the importance of addressing the socioeconomic consequences of TB.

## Teaser

This cross-sectional study examines the types of costs and the occurrence of catastrophic costs associated with the diagnosis and treatment of tuberculosis in India.

## Introduction

India’s National Strategic Plan for Tuberculosis elimination aims to achieve the goal of ending tuberculosis (TB) by 2025, well ahead of the global deadline of 2035.^1^ One of the key aspects of TB burden in India is the extreme poverty often associated with this disease.^2,3^ In spite of the free-of-charge TB diagnostic and treatment services, patients with TB may become trapped in poverty.^4,5^ Extreme levels of socioeconomic difficulty experienced by patients with TB necessitate increased attention toward the World Health Organization’s target of 0 TB-affected families facing catastrophic TB-related costs.^6^ Research pertaining to the economic consequences of TB disease in India is gaining momentum, and substantial research evidence has been generated in recent decades demonstrating that the financial burden on patients with TB is associated with delayed treatment and poor adherence.^7,8,9,10,11^ However, to our knowledge, the occurrence of catastrophic costs associated with TB, as defined by World Health Organization, has not previously been reported from India.

Chennai is one of the most urbanized metropolitan areas in India, with a population of 8 653 521, including approximately 7000 patients with TB who are treated every year under the Revised National Tuberculosis Control Programme (RNTCP).^12^ A prevalence study^13^ conducted during 2010 to 2012 found a TB prevalence rate in Chennai of 259 cases per 100 000, which was higher than the prevalence rates in other parts of the country (207 cases per 100 000). Forty percent of the Chennai population live in slums, are socioeconomically disadvantaged, and have a higher burden of TB compared with nonslum dwellers.^7^ Studies^14^ concerning out-of-pocket expenditures and loss of income associated with TB were conducted in Chennai in 1997, but since then there has been a paucity of evidence about the magnitude of the economic burden associated with TB.

Since 2015, working with private practitioners, the government of India has implemented strategies to reduce costs to patients with TB, such as active case finding, cartridge-based nucleic acid amplification test, and direct benefit transfer (DBT), a scheme to provide Rs 500 (US $7.25 at the 2018 exchange rate of US $1 = Rs 69) per month for nutritional support through electronic transfer into patients’ bank accounts. With this background, it is important to understand the magnitude of and factors associated with catastrophic costs of TB. This study aims to estimate different types of costs and occurrence of catastrophic costs associated with TB diagnosis and treatment.

## Methods

### Setting

The Chennai metropolitan area comprises 54 designated microscopy centers and 36 treatment units, along with 120 urban primary health centers and 90 family welfare centers. In addition, the private health sector consists of approximately 40 major corporate hospitals, approximately 10 000 private practitioners, and approximately 2000 chemists. As per the state planning commission report,^15^ the per capita income of Chennai in 2017 was Rs 66 240. There is also a strong network of nongovernmental and community-based organizations that are engaged in TB control activities.

### Design

A prospective cross-sectional study was conducted that followed up patients with TB registered under the RNTCP. Patients were contacted at 3 different time points: at the initiation of treatment, at the end of the intensive phase (usually at 2 months), and at the end of the continuation phase of treatment (usually at 6 months).

### Ethical Considerations

Before the interview, all respondents received written and oral explanations of the study and signed an informed consent form. Refreshment was provided to the participants during the interview. This study was approved by the Scientific Advisory Committee and Institutional Ethics Committee of the National Institute for Research in Tuberculosis, Indian Council of Medical Research, Chennai. This study follows the Strengthening the Reporting of Observational Studies in Epidemiology (STROBE) reporting guideline.

### Study Population

We sampled patients with pulmonary and extrapulmonary TB consecutively registered under RNTCP between February 2017 and March 2018. Patients with multidrug-resistant TB and patients switching over to category II (ie, patients with sputum smear–positive TB who have relapsed, who have treatment failure, or who are receiving treatment after treatment interruption) from category I (ie, patients with newly diagnosed smear-positive pulmonary TB) were excluded.

### Estimated Sample Size

An earlier study^7^ had observed catastrophic expenditure among 60% of patients with TB in rural areas. Considering this, it was assumed that 40% of urban patients would experience catastrophic costs. With ±5% absolute precision and a 5% level of significance, the estimated sample size was 369. Considering 15% loss at various stages of interview, the sample size was rounded to 450.

### Data Collection

Data were collected from 4 treatment units: Puliyanthope, Elango Nagar, Thondaiyar Pet, and East Cemetery Road (eFigure 1 in the Supplement). Line listing was done from RNTCP laboratory and TB registers. Staff members of RNTCP were given orientation about the study; after obtaining consent, staff met the patient in the health facility or at their residence to collect information.

### Tool for Data Collection

The World Health Organization’s handbook^16^ for TB patient cost surveys was adapted for a longitudinal design. An interview schedule was used to collect information directly from patients, including demographic and socioeconomic characteristics. The questionnaire included detailed information on direct medical costs, direct nonmedical costs, and indirect costs, as well as standard-of-living information.

### Quality Control Measures

The study team was supervised by the study investigators during data collection. Field investigators validated the self-reports by verifying patients’ medical prescription slips, pharmacy bills, and so forth. Further cross-checks were done against the prevailing rates of doctor’s consultation fees, costs for investigations, market price of drugs, and medical bills wherever possible.

### Defined Costing Procedure

All costs were calculated for the entire period from the moment of reported onset of symptoms up to the completion of treatment for 6 to 8 months. All the costs were calculated in terms of Indian rupees (Rs) using the 2018 exchange rate of US $1 = Rs 69.

#### Direct Patient Costs

Direct patient costs included all out-of-pocket expenditures by patients that had been attributed to their TB illness. Consultation fees and money spent on investigations and medicine were classified as direct medical costs. Money spent for transportation to health facilities and costs of food bought during waiting time at the health facility were classified as direct nonmedical costs. These costs were assessed for both the patient as well as persons accompanying the patient.

#### Indirect Patient Costs

Indirect patient costs referred to the costs associated with self-reported work absenteeism and loss of wages associated with illness. These costs included work absenteeism for visits to the health facilities or hospitalization and work absenteeism and loss of wages associated with the inability to work as a result of the TB illness. To quantify the magnitude of loss of income, the number of days absent from work was multiplied by the estimated daily income of the patient or companion.

#### Total Costs

Total costs included all direct and indirect costs incurred before and during treatment. This included both the intensive and continuation phases of treatment.

#### Catastrophic Costs Associated With TB

We used the definition of the World Health Organization for calculating the proportion of the sample experiencing catastrophic costs associated with TB: total costs (indirect and direct combined) exceeding 20% of the household’s annual income.^16^ The total indirect and direct costs of TB were calculated as the sum of (1) out-of-pocket payments for TB diagnosis and treatment made by patients’ households, less any reimbursements; (2) payments associated with the use of TB health services, such as payments for transportation, accommodation, or food, less any reimbursements to the individual who made the payments (ie, guardian or patient); and (3) income losses incurred by both the patient with TB and any accompanying household member, less any social insurance or welfare payment.

### Statistical Analysis

Data were analyzed using SPSS statistical software version 20.0 for PC (IBM). The proportion of households that experienced catastrophic costs was calculated, and a binary response model was used to determine the factors that were significantly associated with catastrophic costs, with 2-tailed *P* < .05 considered statistically significant. A stepwise forward and backward method was used to identify differences in the model’s fitness. The model used demographic and socioeconomic characteristics of the household and lifestyle characteristics of the patient as independent variables. Data analysis was performed from August 2018 to November 2019.

## Results

### Coverage

Overall, 601 patients were registered in 4 centers, and 146 were not interviewed because of nonavailability (11 patients), death (15 patients), migration (8 patients), sickness (2 patients), nontraceability (48 patients), refusal (43 patients), treatment under the 99DOTS Program, a TB treatment program administered by the government of India (13 patients), and other reasons (6 patients). At baseline, a total of 455 patients were enrolled, 423 were interviewed at the end of the intensive phase, and 384 were interviewed at the end of treatment. The final analysis until the end of continuation phase included 384 patients (256 male and 128 female) with follow-up coverage of 91% of patients. Reasons for nonparticipation are shown in the Figure.

**Figure.**
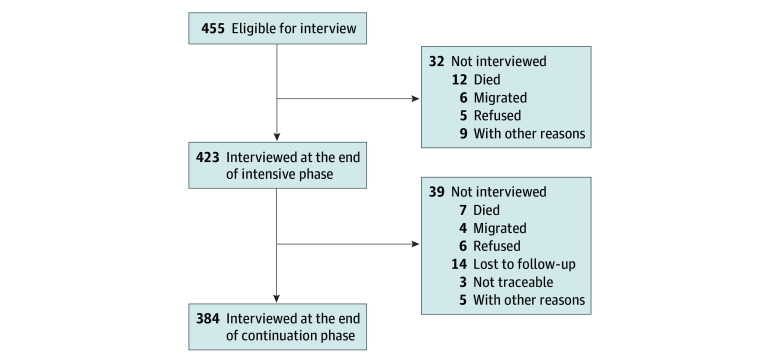
Study Population Flowchart shows reasons that study participants were not interviewed.

### Study Participants’ Characteristics

Table 1 highlights the demographic, socioeconomic, and clinical characteristic of study participants. Two hundred five patients (53%) were aged 19 to 45 years (mean [SD] age, 38.4 [16.0] years), 128 (33%) were female, 72 (19%) were illiterate, 126 (33%) were employed, 116 (30%) earned annually less than Rs 100 000, and 186 (48%) had a single earning member in the family (percentages are based on the 384 patients who were interviewed through the end of the continuation phase of treatment). Only 29% of the population had some form of health security coverage. In terms of lifestyle, tobacco smoking, alcohol use, and tobacco chewing were predominantly reported by male participants. Among male participants, 149 (58%) were alcohol users, 86 (34%) were tobacco smokers, and 27 (11%) were tobacco chewers.

**Table 1.** Demographic, Socioeconomic, Lifestyle, and Clinical Characteristics of the Study Population

Characteristic	Participants, No. (%)		
	Female	Male	Total
Demographic and socioeconomic characteristics			
Age, y			
≤18	32 (25)	14 (5)	46 (12)
19-45	66 (52)	139 (54)	205 (53)
46-65	26 (20)	94 (37)	120 (31)
>65	4 (3)	9 (4)	13 (3)
Education			
Illiterate	27 (21)	45 (18)	72 (19)
Literate	101 (79)	211 (82)	312 (81)
Employment status			
Employed	38 (30)	220 (86)	258 (67)
Unemployed	90 (70)	36 (14)	126 (33)
Annual income, Rs^a^			
<100 000	47 (37)	69 (27)	116 (30)
100 000-200 000	50 (39)	98 (38)	148 (39)
>200 000	31 (24)	89 (35)	120 (31)
Family size, No. of members			
<4	37 (29)	94 (37)	131 (34)
4	35 (27)	84 (33)	119 (31)
>4	56 (44)	78 (30)	134 (35)
Family type			
Joint	41 (32)	63 (25)	104 (27)
Nuclear	87 (68)	193 (75)	280 (73)
No. of earning members			
≤1	68 (53)	118 (46)	186 (48)
>1	60 (47)	138 (54)	198 (52)
Lifestyle indicators			
Alcohol users			
No	127 (99)	107 (42)	234 (61)
Yes	1 (1)	149 (58)	150 (39)
Tobacco smoking			
No	128 (100)	170 (66)	298 (78)
Yes	0	86 (34)	86 (22)
Tobacco chewing			
No	126 (98)	229 (89)	355 (92)
Yes	2 (2)	27 (11)	29 (8)
Clinical characteristics			
Type of tuberculosis			
Pulmonary smear negative	32 (25)	36 (14)	68 (18)
Pulmonary smear positive	57 (45)	177 (69)	234 (61)
Extrapulmonary	39 (30)	43 (17)	82 (21)
Treatment regimen			
Category I^b^	66 (52)	181 (71)	247 (64)
Category II^c^	52 (41)	51 (20)	103 (27)
Non–directly observed therapies	10 (8)	24 (9)	34 (9)
HIV status			
Positive	0	3 (1)	3 (1)
Negative	106 (83)	208 (81)	314 (82)
Unknown	22 (17)	45 (18)	67 (17)

^a^ In 2018, US $1 = 69 Indian rupees (Rs).

^b^ Patients with newly diagnosed smear-positive pulmonary tuberculosis.

^c^ Patients with sputum smear–positive tuberculosis who have relapsed, who have treatment failure, or who are receiving treatment after treatment interruption.

Among patients interviewed, 234 (61%) had pulmonary smear-positive TB. This proportion was higher among male participants compared with female participants (69% vs 45%; difference, 24%; 95% CI, 13%-35%; *P* < .001). Overall, 82 patients (21%) had extrapulmonary TB, and the proportion was higher among female participants compared with male participants (30% vs 17%; difference, 13%; 95% CI, 4%-23%; *P* = .003).

### Household Costs for TB Diagnosis and Treatment

In terms of costs incurred for diagnosis of TB, the overall mean (SD) cost was Rs 11 779 (Rs 18 972) (median [range], Rs 4565 [Rs 1-151 770]), of which a mean (SD) of Rs 4118 (Rs 11 457) (median [range], Rs 885 [Rs 0-151 770]) was direct costs and a mean (SD) of Rs 7662 (Rs 15 164) (median [range], Rs 0 [Rs 0-120 400]) was indirect costs. With respect to costs incurred during treatment, the mean (SD) overall costs were Rs 19 364 (Rs 36 269) (median [range], Rs 3015 [Rs 0-313 000]) of which Rs 3367 (Rs 12 776) (median [range], Rs 1070 [Rs 0-219]) were direct cost and Rs 15 996 (Rs 33 778) (median [range], Rs 0 [Rs 0-305 000]) were indirect costs (eTable in the Supplement).

Both during diagnostic and treatment phases, the indirect cost incurred was higher than the direct cost. During diagnosis, more than 50% of patients did not incur indirect costs and more than 80% of patients spent less than Rs 1000 as direct costs. During the treatment phase, more than 20% of the patients spent less than Rs 30 000 as indirect costs (eFigure 2 in the Supplement).

The different components of costs incurred by patients during treatment are shown in Table 2. We found that 190 patients (49%) incurred travel costs, 85 (22%) incurred hospitalization costs, and 65 (17%) incurred food costs. Indirect costs were incurred by 132 patients themselves (34%) and by 23 patients’ guardians (6%). Furthermore, we found that 30% of the patients borrowed money for their treatment. It was also observed that 41% of the study population accessed private hospitals as the first point of care and that they incurred higher direct costs for diagnosis compared with those who accessed government hospitals (Rs 7090 vs Rs 2040; difference, Rs 5050; 95% CI, Rs 2766-7332; *P* < .001).

**Table 2.** Components of Costs Incurred by Patients During Treatment

Type of Cost	Cost Incurred, Patients, No. (%)^a^		Cost, Rs, Mean (SD)^b^	Cost, Rs, Median (Range)^b^
	Yes	No		
Direct cost				
Travel	190 (49)	194 (51)	58 (96)	0 (0-800)
Food	65 (17)	319 (83)	15 (49)	0 (0-600)
Follow-up	12 (3)	372 (97)	3 (25)	0 (0-360)
Hospitalization	85 (22)	299 (78)	1741 (7309)	0 (0-91 000)
Escort	37 (10)	347 (90)	190 (766)	0 (0-7000)
Visitors	30 (8)	354 (92)	28 (194)	0 (0-3000)
Indirect cost				
Patient	132 (34)	252 (66)	13 604 (28 776)	0 (0-215 682)
Guardian	23 (6)	361 (94)	2089 (17 690)	0 (0-270 000)
Hospitalization escort	21 (5)	363 (95)	255 (1997)	0 (0-35 000)

^a^ Data are shown for 384 patients.

^b^ In 2018, US $1 = 69 Indian rupees (Rs).

### Factors Associated With Catastrophic Costs

We found that 31% of patients experienced catastrophic costs associated with TB. Univariate analysis identified a wide range of factors associated with an increased risk of incurring catastrophic costs (Table 3). Multivariate logistic regression analysis found that unemployment (adjusted odds ratio, 0.2; 95% CI, 0.1-0.5; *P* < .001) and higher annual household income (Rs 1-200 000, adjusted odds ratio, 0.4; 95% CI, 0.2-0.7; *P* = .004; greater than Rs 200 000, adjusted odds ratio, 0.2; 95% CI, 0.1-0.5; *P* < .001) were associated with a decreased risk of incurring catastrophic costs.

**Table 3.** Factors Associated With Catastrophic Costs Associated With Tuberculosis

Variable	Catastrophic Costs Incurred, Participants, No. (%)		Unadjusted Analysis		Adjusted Analysis	
	No	Yes	OR (95% CI)	*P* Value	OR (95% CI)	*P* Value
Sex						
Female	96 (36)	32 (27)	1 [Reference]		1 [Reference]	
Male	168 (64)	88 (73)	1.6 (0.9-2.5)	.06	0.7 (0.3-1.4)	.26
Age, y						
≤18	40 (15)	6 (5)	1 [Reference]		1 [Reference]	
19-45	136 (52)	69 (58)	3.4 (1.4-8.4)	.008	1.8 (0.6-5.5)	.28
46-65	78 (30)	42 (35)	3.6 (1.4-9.2)	.007	1.7 (0.5-5.3)	.36
>65	10 (4)	3 (3)	2.0 (0.4-9.4)	.38	2.3 (0.4-12.7)	.34
Type of tuberculosis						
Smear negative	50 (19)	18 (15)	1 [Reference]		1 [Reference]	
Smear positive	158 (60)	76 (63)	1.3 (0.7-2.4)	.35	1.5 (0.5-2.5)	.71
Extrapulmonary	56 (21)	26 (22)	1.3 (0.6-2.6)	.48	1.1 (0.4-2.7)	.85
Treatment regimen						
Category I^a^	174 (66)	73 (61)	1 [Reference]		1 [Reference]	
Category II^b^	71 (27)	32 (27)	1.1 (0.7-1.8)	.78	2.0 (0.9-4.6)	.10
Non–directly observed therapies	19 (7)	15 (13)	1.9 (0.9-3.9)	.09	2.1 (0.9-5.0)	.08
Education						
Literate	219 (83)	93 (78)	1 [Reference]		1 [Reference]	
Illiterate	45 (17)	27 (23)	1.4 (0.8-2.4)	.21	1.4 (0.7-2.6)	.33
Employment status						
Employed	159 (60)	99 (83)	1 [Reference]		1 [Reference]	
Unemployed	105 (40)	21 (18)	0.3 (0.2-0.5)	<.001	0.2 (0.1-0.5)	<.001
Annual income, Rs^c^						
<100 000	65 (25)	51 (43)	1 [Reference]		1 [Reference]	
100 000-200 000	104 (39)	44 (37)	0.5 (0.3-0.9)	.02	0.4 (0.2-0.7)	.004
>200 000	95 (36)	25 (21)	0.3 (0.2-0.6)	<.001	0.2 (0.1-0.5)	<.001
Family size, No. of members						
<4	83 (31)	48 (40)	1 [Reference]		1 [Reference]	
4	78 (30)	41 (34)	0.9 (0.5-1.5)	.72	1.3 (0.7-2.3)	.44
>4	103 (39)	31 (26)	0.5 (0.3-0.9)	.02	1.0 (0.5-1.9)	.94
Family type						
Joint	80 (30)	24 (20)	1 [Reference]		1 [Reference]	
Nuclear	184 (70)	96 (80)	1.7 (1.1-2.9)	.04	1.5 (0.8-2.9)	.24
No. of earning members						
1	120 (45)	66 (55)	1 [Reference]		1 [Reference]	
>1	144 (55)	54 (45)	0.7 (0.4-1.1)	.08	1.2 (0.6-2.2)	.61
Tobacco chewing						
No	247 (94)	108 (90)	1 [Reference]		1 [Reference]	
Yes	17 (6)	12 (10)	1.6 (0.7-3.5)	.22	1.1 (0.5-2.5)	.88
Tobacco smoking						
No	215 (81)	83 (69)	1 [Reference]		1 [Reference]	
Yes	49 (19)	37 (31)	2.0 (1.2-3.2)	.008	1.5 (0.8-2.8)	.21
Alcohol use						
No	176 (67)	58 (48)	1 [Reference]		1 [Reference]	
Yes	88 (33)	62 (52)	2.1 (1.4-3.3)	.001	1.4 (0.8-2.6)	.28

Abbreviation: OR, odds ratio.

^a^ Patients with newly diagnosed smear-positive pulmonary tuberculosis.

^b^ Patients with sputum smear–positive tuberculosis who have relapsed, who have treatment failure, or who are receiving treatment after treatment interruption.

^c^ In 2018, US $1 = 69 Indian rupees (Rs).

## Discussion

In this prospective cross-sectional cohort study of patients with TB who were registered under RNTCP, 31% of patients experienced catastrophic costs associated with TB. In spite of the provision of completely free-of-charge TB diagnostic and treatment services under RNTCP, our findings highlight that a significant proportion of patients faced economic consequences associated with TB. Our findings reemphasize that accessing ostensibly free TB diagnostic and treatment services can be expensive for poor patients with TB. High health care costs significantly reduce households’ resilience against strenuous expenditure on basic necessities, such as food, housing, and children’s education.^17^ The cost was higher than what has been reported from other parts of the country,^18^ although those reports used different methods.

Catastrophic costs are more likely to be incurred by patients in lower socioeconomic segments. Families will be stressed, leading to loss of savings and assets and the incurring of debt.^19^ To reduce the household economic burden associated with TB, provisions such as transportation support, drugs for symptom relief, special food, and compensation for loss of income could be implemented.^4,14^ These findings underscore the need to address broader socioeconomic consequences experienced by patients and their families.^20^ In our study area, although the DBT scheme has already been implemented, the level of costs is still much higher than the DBT allowance for most patients. An allowance of Rs 500 per month adds up to 3000 over the course of 6 months, which is much lower than the mean total of Rs 30 000 in our study. This fact highlights that, although DBT would help to provide financial protection to some extent, it still would not be enough to provide sufficient compensation for most patients.

The costs incurred during the diagnostic phase are lower than those incurred during the treatment phase. Earlier findings showed that costs incurred during the diagnostic phase were higher than those incurred during the treatment phase.^7^ This fact could be attributed to the improved awareness about TB symptoms, which prevents people from accessing multiple practitioners for diagnosis. Also, increased public diagnostic facilities may have enabled earlier and easier diagnosis of TB.^13^ Active case finding (ie, door to door) for early and increased case detection could substantially reduce patient cost for diagnosis.^21^

Reasons for increased costs during treatment could be attributed, in part, to additional nutrition intake and physical rest required by patients. Also, improved adherence increases travel frequency and job absenteeism, which could increase treatment costs. We found that 49% of the study patients incurred travel costs during treatment. This finding suggests that there is a need to provide treatment very close to the patient’s residence or travel reimbursement or free bus passes. Family members also experienced work absenteeism associated with care demands for the patient. This is substantiated by the fact that 33% of our patients were employed, 48% were the single earner in their family, and 69% were male patients with pulmonary TB. Furthermore, our findings suggest that catastrophic costs of TB were similar among literate and illiterate patients. This necessitates the provision of additional financial protection through insurance schemes or financial support during treatment period.

We found that only 29% of the population had some form of health security coverage. In addition to the DBT and other support schemes, there is an urgent need to incorporate innovative financial protection tools through intersectoral and interprogram collaborations and private sector and civil societies’ involvement.^21,22^ Findings from an innovative health financing package implemented and tested in Chhattisgarh showed positive outcomes in addressing the huge costs incurred for diagnosis and treatment of drug resistance TB among patients in lower socioeconomic strata.^23^ Such financial protection packages for patients in lower socioeconomic strata could be expanded for patients with drug-sensitive TB as well.^24,25^

### Limitations

This study has a few limitations. The data collected from patients with TB were self-reported, which may be subject to recall bias. Indirect costs for patients who were not working, such as students, those who work at home, and those who were unemployed, may have been underestimated, which might have resulted in unemployed patients being less likely to have experienced catastrophic costs. This finding is generalizable where similar low socioeconomic status is prevalent.

## Conclusions

In this study, approximately one-third of the patients with TB registered under RNTCP in Chennai experienced catastrophic costs. We identified a large gap between the costs incurred and the present supportive financial protection interventions. There is an urgent need to develop and implement new financial protection packages and necessary social support for patients with TB to address their basic needs.

## Notes

Supplement. **eTable.** Direct, Indirect and Total Costs in Rupees for TB Diagnosis, Treatment Incurred by Patients Taking Treatment Under RNTCP, in Chennai **eFigure 1.** Study Area **eFigure 2.** Overall Costs (Direct, Indirect) for Diagnosis and Treatment
