# Supplementary material for: Association of Tuberculosis With Household Catastrophic Expenditure in South India
Source: JAMA Netw Open. 2020 Feb 12;3(2):e1920973. doi: 10.1001/jamanetworkopen.2019.20973 (PMC11845097; doi:10.1001/jamanetworkopen.2019.20973)
Supplement: Supplement. — eTable. Direct, Indirect and Total Costs in Rupees for TB Diagnosis, Treatment Incurred by Patients Taking Treatment Under RNTCP, in Chennai eFigure 1. Study Area eFigure 2. Overall Costs (Direct, Indirect) for Diagnosis and Treatment [file jamanetwopen-e1920973-s001.pdf]

Supplementary Online Content

Muniyandi M, Thomas BE, Karikalan N, et al. Association of tuberculosis with household catastrophic expenditure in South India. *JAMA Network Open*. 2020;3(2):e1920973. doi:10.1001/jamanetworkopen.2019.20973

eTable. Direct, Indirect and Total Costs in Rupees for TB Diagnosis, Treatment Incurred by Patients Taking Treatment Under RNTCP, in Chennai

eFigure 1. Study Area

eFigure 2. Overall Costs (Direct, Indirect) for Diagnosis and Treatment

This supplementary material has been provided by the authors to give readers additional information about their work.

© 2020 Muniyandi M et al. *JAMA Network Open.*

eTable. Direct, Indirect and Total Costs in Rupees for TB Diagnosis, Treatment Incurred by Patients Taking Treatment Under RNTCP, in Chennai

	Mean	SD	Median	Range
**Direct**				
Diagnosis	4118	11457.6	885	0-151770
Treatment	3367	12776	1070	0-219.4
Total	7485	21182	2425	0-312000
**Indirect**				
Diagnosis	7662	15164	0.0	0-120400
Treatment	15996	33778	0.0	0-305000
Total	23658	43764	2841	0-309182
**Overall**				
Diagnosis	11779	18972	4565	0-151770
Treatment	19364	36269	3015	0-313000
Total	31143	48977	10320	0-317890

© 2020 Muniyandi M et al. *JAMA Network Open.*

eFigure 1. Study Area

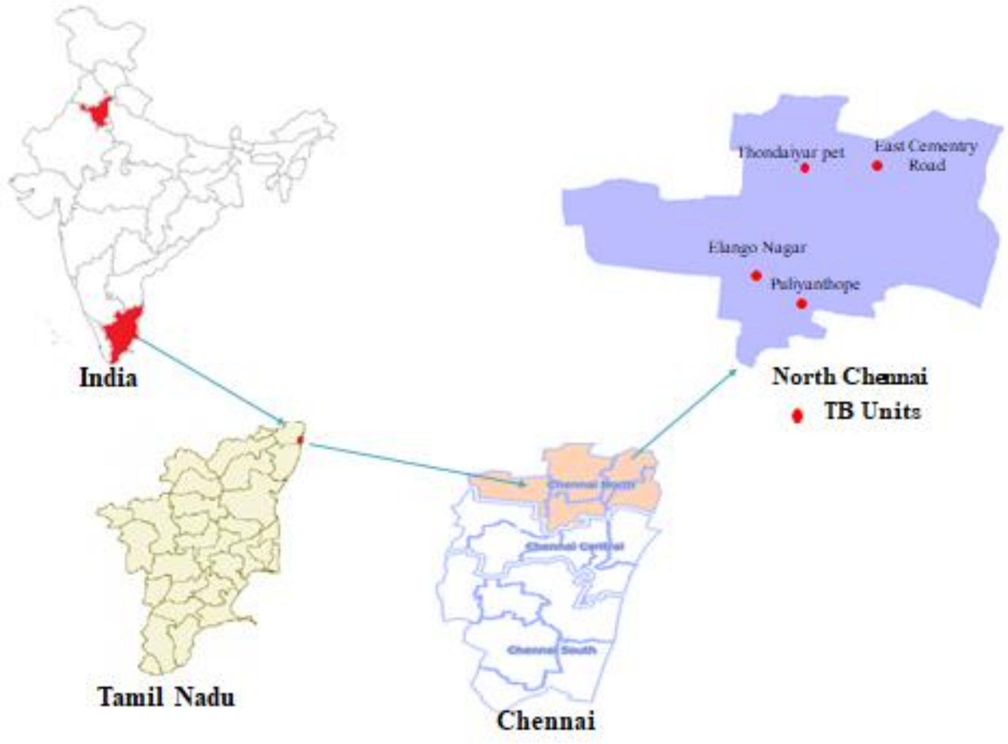

© 2020 Muniyandi M et al. *JAMA Network Open.*

eFigure 2. Overall Costs (Direct, Indirect) for Diagnosis and Treatment

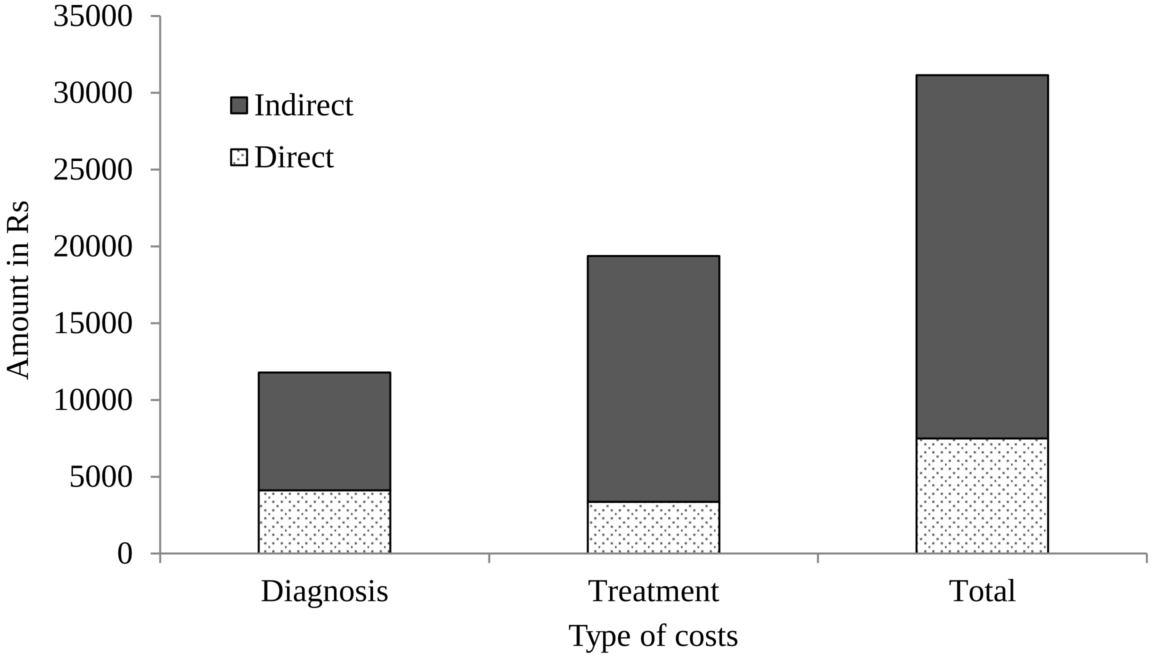

© 2020 Muniyandi M et al. *JAMA Network Open.*
